# Estimating the Impact of Daily Weather on the Temporal Pattern of COVID-19 Outbreak in India

**DOI:** 10.1007/s41748-020-00179-1

**Published:** 2020-09-17

**Authors:** Amitesh Gupta, Biswajeet Pradhan, Khairul Nizam Abdul Maulud

**Affiliations:** 1 Remote Sensing and GIS Department, JIS University, Agarpara, Kolkata, India; 2 Centre for Advanced Modelling and Geospatial Information Systems (CAMGIS), School of Information, Systems and Modelling, Faculty of Engineering and IT, University of Technology Sydney (UTS), Sydney, Australia; 3 Earth Observation Centre, Institute of Climate Change (IPI), Universiti Kebangsaan Malaysia (UKM), 43600 UKM Bangi, Selangor Malaysia; 4 Department of Civil Engineering, Faculty of Engineering and Built Environment, Universiti Kebangsaan Malaysia, 43600 UKM Bangi, Selangor Malaysia

**Keywords:** COVID-19, Weather, Temporal trend, India

## Abstract

The COVID-19 pandemic has spread obstreperously in India. The increase in daily confirmed cases accelerated significantly from ~ 5 additional new cases (ANC)/day during early March up to ~ 249 ANC/day during early June. An abrupt change in this temporal pattern was noticed during mid-April, from which can be inferred a much reduced impact of the nationwide lockdown in India. Daily maximum (*T*_Max_), minimum (*T*_Min_), mean (*T*_Mean_) and dew point temperature (*T*_Dew_), wind speed (WS), relative humidity, and diurnal range in temperature and relative humidity during March 01 to June 04, 2020 over 9 major affected cities are analyzed to look into the impact of daily weather on COVID-19 infections on that day and 7, 10, 12, 14, 16 days before those cases were detected (i.e., on the likely transmission days). Spearman’s correlation exhibits significantly lower association with WS, *T*_Max_, *T*_Min_, *T*_Mean_, *T*_Dew_, but is comparatively better with a lag of 14 days. Support Vector regression successfully estimated the count of confirmed cases (*R*^2^ > 0.8) at a lag of 12–16 days, thus reflecting a probable incubation period of 14 ± 02 days in India. Approximately 75% of total cases were registered when *T*_Max_, *T*_Mean_, *T*_Min_, *T*_Dew_, and WS at 12–16 days previously were varying within the range of 33.6–41.3 °C, 29.8–36.5 °C, 24.8–30.4 °C, 18.7–23.6 °C, and 4.2–5.75 m/s, respectively. Thus, we conclude that coronavirus transmission is not well correlated (linearly) with any individual weather parameter; rather, transmission is susceptible to a certain weather pattern. Hence multivariate non-linear approach must be employed instead.

## Introduction

In human history, it is apparent that pathogens have caused devastating consequences in social wellbeing and economies (Briz-Redón and Serrano-Aroca 2020). The recent novel coronavirus disease (COVID-19) is one prominent example of such a disastrous event that has grasped the world. The earliest outbreak of COVID-19 caused by Severe Acute Respiratory Syndrome CoronaVirus-2 (SARS-CoV-2) happened in Wuhan, Hubei Province, China during the late December, 2019, (Guan et al. 2020; Wu and McGoogan 2020; Zhu et al. 2019; Zu et al. 2020). Because of human-to-human transmissibility of the virus by contact, droplets and fomites (Wang et al. 2020a, b), the transmission of this disease has become progressively more unpredictable and populations have become more vulnerable. Considering the rapid spread of the virus, the World Health Organization (WHO) declared an international public health emergency on January 30, 2020, and later on March 11, 2020, WHO declared this disease to be a global pandemic, due to the exponential surge in the total number of infections. Up to June 04, 2020, a total of 6,697,418 cases have been affirmed with 5.85% of these resulting in deaths worldwide (https://www.worldometers.info/coronavirus). Despite the fact that India registered its first case on January 29, 2020, the real outbreak occurred from March 02, 2020 onwards, and as of June 04, 2020, a total of 226,722 cases have been confirmed; however, the death rate (2.81%) is much lower than in the rest of the world.

Clinical investigations of COVID-19 identified respiratory droplets as the most common agent of infection (Ge et al. 2013; Huang et al. 2020) and the symptoms are also quite analogous to other coronavirus diseases such as MERS and SARS (Holshue et al. 2020; Perlman 2020; Tan et al. 2005; Wang et al. 2020c). WHO also reported that the SARS-CoV-2 virus initially causes respiratory disease, presents as a wide range of illness from asymptomatic or mild through to severe disease and death. Thus, the COVID-19 disease has close similarities in its presentation to influenza (https://www.who.int/westernpacific/news/q-a-detail/).

Environmental factors, such as daily weather and long-term climatic conditions, may affect the epidemiological dynamics of this type of infectious disease (Dalziel et al. 2018; Yuan et al. 2006). Daily air temperature and relative humidity may impact on the transmission of coronavirus by affecting the persistence of the viral infections within its transmission routes (Casanova et al. 2010). A few studies accounting for climate and weather conditions found that these factors considerably affect the spatial distribution of the disease, along with its incubation period (Bedford et al. 2015; Lemaitre et al. 2019; Sooryanarain and Elankumaran 2015). Many years ago, Bull (1980) was the first to report that the mortality rate of pneumonia is intimately associated with changes in weather conditions. Other studies have revealed that among different climatic variables, air temperature affects influenza epidemics mostly in tropical regions (Tamerius et al. 2013), whereas the mid-latitude temperate regions experience influenza epidemics mostly during winter months (Bedford et al. 2015; Sooryanarain and Elankumaran 2015). Nevertheless, the response of COVID-19 transmission to weather patterns remains debatable, since studies carried out in different countries suggested an existing correlation between weather and the COVID-19 pandemic (Ficetola and Rubolini 2020; Liu et al. 2020; Ma et al. 2020; Oliveiros et al. 2020; Qi et al. 2020; Tosepu et al. 2020). Contradictorily, a few studies have reported that meteorological observations are not correlated with the outbreak pattern (Jamil et al. 2020; Mollalo et al. 2020; Shi et al. 2020; Xie and Zhu 2020). Studies carried out by (Wang et al. 2020a, b) suggested that the spread of disease would decrease with an increase in temperature. Based on the USA model, a reduction of transmission in warmer conditions had been predicted for India (Gupta et al. 2020a). However, in view of the long-term climate record, it was found that comparatively hot areas in India are possibly going to be more affected by this disease (Gupta et al. 2020b). On the basis of regional data for several provinces in India, Goswami et al. (2020) reported on the inconsistency of the weather-infection interrelationship in India. Besides, the incubation period of COVID-19 may also vary spatially. The WHO reported an incubation period of 2–10 days for COVID-19 based on worldwide observation (World Health Organization 2020) while the National Health Commission in China had initially estimated an incubation period of 10–14 days for China (https://www.aljazeera.com/news/2020/01/chinas-national-health-commission-news-conference-coronavirus-200126105935024.html). The Centres for Disease Control and Prevention in United States of America estimate an incubation period of 2–14 days (https://www.cdc.gov/coronavirus/2019-ncov/symptoms-testing/symptoms.html). On the other hand, some studies reported an incubation period of around 20 days (Bai et al. 2020; Guan et al. 2020).

COVID-19 has already made a significant indirect impact through reduction in anthropogenic activities on several environmental aspects in the Indian context (Gupta et al. 2020c), however, only a few studies have investigated the impact of daily weather on COVID-19 transmission nationwide, and since the incubation period of this disease in India is also not mentioned anywhere to date, there is a need for a comprehensive study about the impact of weather patterns on COVID-19 transmission in the Indian scenario. Thus, the present study is aimed at understanding the temporal patterns of the outbreak, any abrupt changes and the influence of daily weather conditions on the daily count of infected cases in India. We have also attempted to estimate the incubation period of COVID-19 based on five different time-frames: precisely on the day of the case detected, and with leads of 7, 10, 12, 14, and 16 days prior to the case detection.

## Data and Methodology

### Data Collection

**Fig. 1:**
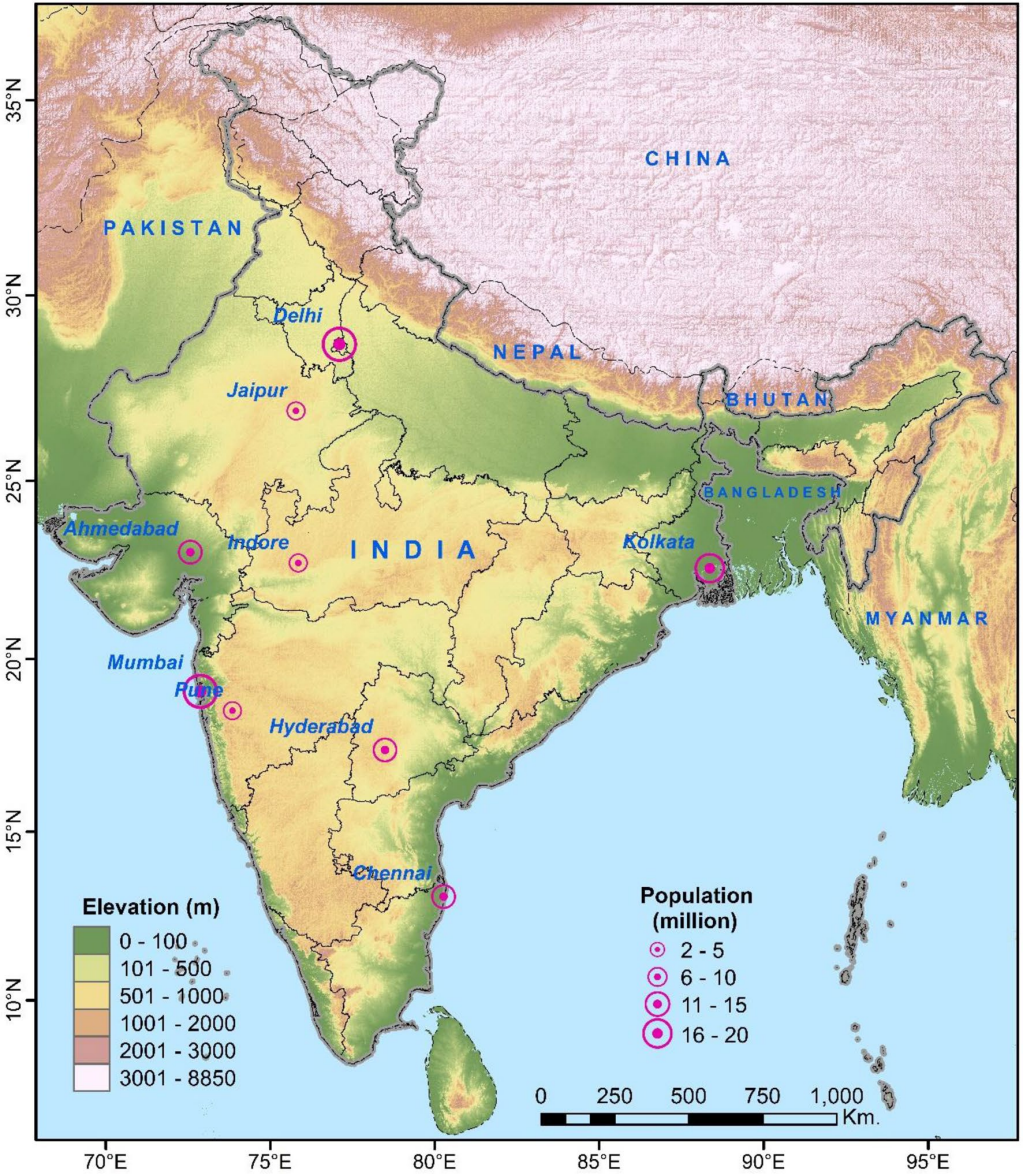
Location of the selected cities in India along with the total population of those cities

**Fig. 2:**
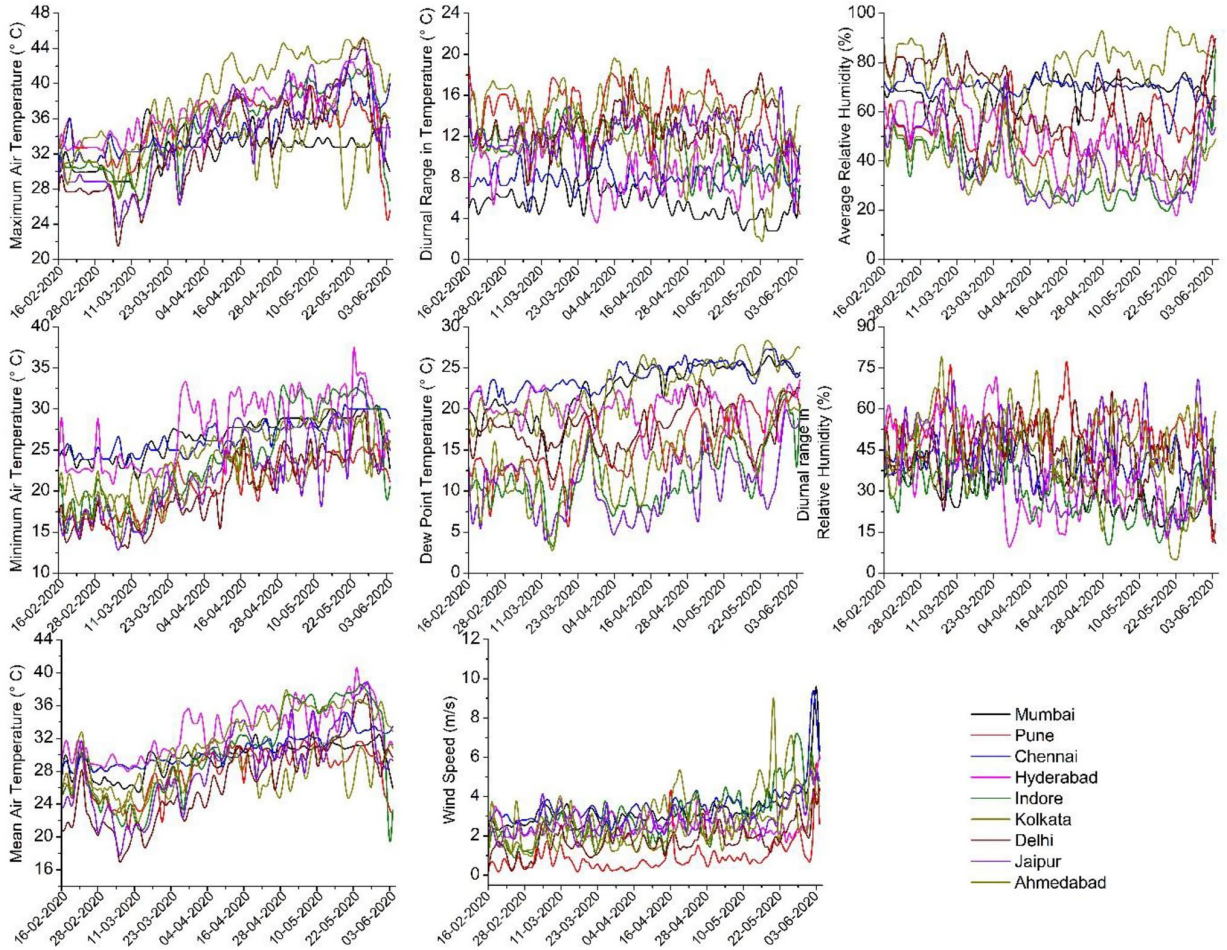
Pattern of daily weather over the selected cities in India

India, the largest country in South Asia, extends from 6° N to 38° N, and from 68° E to 98° E, comprising a land area of 3.287 million sq. km. with a total population of more than 1.2 billion (Census of India Website 2011). The data of daily COVID-19 cases were collected from the official website of the Ministry of Health of India (https://www.mohfw.gov.in). Among a total of 725 districts in India, 618 districts have reported multiple confirmed cases. Several studies have reported that the disease spreads faster in the cities where population density is very high (Casanova et al. 2010; Ahmadi et al. 2020; Bonasera and Zhang 2020; Kang et al. 2020; Rocklöv and Sjödin 2020). Thus, among 53 ‘million cities’ (where the total population is more than one million) in India, 9 cities have been selected for this study, from where more than 79% of the total cases in India have been reported up to June 4, 2020 (Fig. 1). The daily weather data were collected from https://www.wunderground.com. Figure 2 shows the prevailing daily weather conditions in terms of maximum, minimum and mean temperature of air, diurnal range in air temperature, dew point temperature, average relative humidity, diurnal range in relative humidity, and wind speed, in those cities. Since all the selected cities are located in different bio-climatic zones having different temperature characteristics (Gupta 2017), the variations in meteorological observations will also help to identify how spatially varying weather conditions influence the pattern of COVID-19 transmission in India.

### Mann–Kendall Test

The nonparametric Mann–Kendall (MK) method (Kendall 1975; Mann 1945) was applied to the daily data of COVID-19 confirmed cases during March 01 to June 04, 2020 to detect statistically significant trends. The MK test takes as the null hypothesis (*H*_0_) that there is no trend in the count of confirmed cases of infections; while the alternate hypothesis (*H*_1_) is that there is a trend (increasing or decreasing) over time. The mathematical expressions for calculating MK Statistics *S*, *V*(*S*) and standardized test statistics *Z* are as follows:

1 $$S = \mathop \sum \limits_{i = 1}^{T - 1} \mathop \sum \limits_{j = i + 1}^{T} {\text{sgn}} (X_{j} - X_{i} ),$$

2 $${\text{sgn}} \left( {X_{j} - X_{i} } \right) = \left\{ {\begin{array}{*{20}c} { + 1} &\quad {{\text{if}} \; \left( {X_{j} - X_{i} } \right) > 0} \\ 0 & \quad {{\text{if}} \left( {X_{j} - X_{i} } \right) = 0} \\ { - 1} & {{\text{if}} \; \left( {X_{j} - X_{i} } \right) < 0} \\ \end{array} } \right\},$$

3 $$V \left( S \right) = \frac{1}{18}\left[ {T\left( {T - 1} \right)\left( {2T + 5} \right) - \mathop \sum \limits_{p = 1}^{q} t_{p} p\left( {p - 1} \right)\left( {2p + 5} \right)} \right],$$

4 $$Z = \left\{ {\begin{array}{*{20}l} {\frac{S - 1}{{\sqrt {{\text{VAR}} \left( S \right)} }}} \hfill &\quad {{\text{if}} \;S > 0} \hfill \\ 0 \hfill &\quad {{\text{if}} \;S = 0} \hfill \\ {\frac{S + 1}{{\sqrt {{\text{VAR}} \left( S \right)} }}} \hfill & {{\text{if}} \;S < 0} \hfill \\ \end{array} } \right\},$$

where, *X*_*i*_ and *X*_*j*_ are the daily observations, *T* is the length of the time series, ${t}_{p}$ is the number of ties for the *p*th value. Positive *Z* values designate an increasing trend and negative *Z* values signpost a negative trend. For |*Z*|> *Z*_1−α/2_, *H*_1_ is accepted with rejection of *H*_0_, considering the critical value of *Z*_1−α/2_ to be 1.96 for a *p* value of 0.05.

The statistic *S* is closely related to the Kendall’s τ which is given by:

$$\tau = \frac{S}{D}$$

where

5 $$D = \left[ {\frac{1}{2}T\left( {T - 1} \right) - \frac{1}{2}\mathop \sum \limits_{p = 1}^{q} t_{p} p\left( {p - 1} \right)} \right]^{\frac{1}{2}} \left[ {\frac{1}{2}p\left( {p - 1} \right)} \right]^{\frac{1}{2}} .$$

### Sen’s Slope Estimator

Sen’s slope (Sen 1968) is widely employed to estimate the magnitude of trends.

6 $$d_{i} = {\text{Median}} \left[ {\frac{{x_{j} - x_{k} }}{j - k}} \right] \quad {\text{for all}} \;j > k,$$

where $d$ is the slope, *x*_*j*_ and *x*_*k*_ represent the corresponding data values at time *j* and *k*, (1 ≤ *k* < *j* ≤ *n*), *n* is the number of the variables.

7 $$Q_{i} = \left\{ {\begin{array}{*{20}l} {d_{{\left( {n + 1} \right)/2}} } & {{\text{if}} \;n\; {\text{is odd}}} \\ {\frac{1}{2}\left( {d_{n/2} + d_{{\left( {n + 2} \right)/2}} } \right)\frac{1}{2}\left( {d_{n/2} + d_{{\left( {n + 2} \right)/2}} } \right)} & {{\text{if}}\;n\; {\text{is even}}} \\ \end{array} } \right\}.$$

A positive *Q*_*i*_ value denotes an increasing trend; a negative *Q*_*i*_ value signifies a decreasing trend.

In this study, the MK test and Sen’s Slope Estimator were implemented to investigate the trend of daily transmission over selected cities as well as all over the whole country. This helped to establish whether the temporal pattern of transmission varied in different cities with respect to the countrywide pattern or not.

### Pettitt Test

Originally developed by (Pettitt 1979), the non-parametric Pettitt test is an effective method of identifying the change in the temporal trend in any time-series, because of its sensitivity to breaks in the middle of temporal records (Gao et al. 2011; Hänsel et al. 2016; Jaiswal et al. 2015; Mallakpour and Villarini 2016; Wijngaard et al. 2003). In this method, *S* is evaluated for all random variables from 1 to *T*; then the most prominent change point is determined as that where the value of *|S|* found to be largest:

8 $$K_{T} = {\text{ max}}_{{1{ } \le { }t{ } < {\text{ T}}}} \left| S \right|.$$

At a particular time *t*, the change point is detected when *K*_*T*_ is clearly different from zero at any particular level, where the significant level is estimated by:

9 $$p = 2 \times \exp \left( {\frac{{ - 6K_{T}^{2} }}{{T^{2} + T^{3} }}} \right).$$

The change point can be evaluated as statistically significant only when the estimated *p* value becomes less than the pre-assigned significance level, i.e., *α*.

### Growth Rate

Growth rate denotes the magnitude of alteration of any particular variable within a definite time period. Here, growth rate between March 01 and June 04, 2020 for the overall country and for each selected city was calculated using following formula:

10 $${\text{Growth rate}}\; \left( \% \right) = \left\{ {\left( {\frac{{{\text{NF}}}}{{{\text{NE}}}}} \right)^{{{\raise0.7ex\hbox{$1$} \!\mathord{\left/ {\vphantom {1 n}}\right.\kern-\nulldelimiterspace} \!\lower0.7ex\hbox{$n$}}}} - 1} \right\} \times 100.$$

Here, $\mathrm{NF}$ refers to the number of COVID-19 cases recorded on the 1st day of record,$\mathrm{NE}$ refers to the number of COVID-19 cases recorded on the last day of the study period (June 04, 2020), and $n$ refers to the number of days between the first day of COVID-19 case detection and the last day of the study period.

### Doubling Time

The doubling time denotes the time taken for a count to be doubled. Here doubling time for the overall country and for each selected city was calculated using following formula:

11 $${\text{Doubling time}} = \frac{n \times \ln \left( 2 \right)}{{\ln \left( {{\text{NE}}/{\text{NF}}} \right)}}.$$

### Spearman's Correlation Test

Spearman's rank correlation coefficient (*r*_s_) calculates the association between the number of daily new cases and other input parameters. It summarizes how well the association between daily transmission and weather parameters can be quantified. The coefficient can be calculated via following equation:

12 $$r_{{\text{s}}} = 1 - 6\frac{{\sum d_{i}^{2} }}{{n\left( {n^{2} - 1} \right)}},$$

where, *n* represents the number of alternatives, and *d*_*i*_ is the difference between the ranks of two parameters.

All the above mentioned statistics were based on a 95% confidence level.

### Support Vector Machine

Support Vector Machine (SVM) is an extensively utilized machine learning technique. It is performed on the basis of statistical auto-adaptation and the structural risk minimization principle (Tien Bui et al. 2012). By creating a hyper-plane, the nonlinearity in the input dataset is reshaped into a linear entity (Jebur et al. 2014). The key factor behind this data transformation is a kernel function. Using the assigned training dataset, SVM puts the original input into a higher dimensional feature space, then finds the supreme fringe of separation among the observations, and constructs a hyper-plane at the centre of that extreme margin (Marjanović et al. 2011). Support vectors are nothing but the nearest training points to the produced hyper plane. Thus, this model adapts itself with new input observations, creates a hyper-plane and identifies the support vectors and thereafter acts on the input variables of the testing dataset to estimate the predicted variable. Further insights about how the mathematical computations and procedures work in SVM can be found in several papers such as Tien Bui et al. (2012), Pradhan (2013), Tehrany et al. (2014), Tehrany et al. (2015). However, the accuracy of estimation depends on the kernel type selected during the training of the model (Yao et al. 2008). The radial basis function (RBF) kernel produces more exact results and is preferred over the linear, polynomial and sigmoid kernels, due to its higher capability in interpolation (Song et al. 2011).

In the present study, the log-transformed values of daily COVID-19 cases were estimated using several daily weather parameters, along with the elevation and population of those cities (Eq. 13).

13 $$\ln \left( {{\text{NC}}} \right) = {\text{SVM}}\left( {T_{{{\text{Max}}}} + T_{{{\text{Min}}}} + T_{{{\text{Mean}}}} + T_{{{\text{range}}}} + T_{{{\text{Dew}}}} + H_{{{\text{Avg}}}} + H_{{{\text{range}}}} + {\text{WS}} + {\text{Ele}} + {\text{Pop}}} \right),$$

where, $\mathrm{NC}$ is the number of new confirmed case, ${T}_{\mathrm{Max}}$ is maximum air temperature (°C), ${T}_{\mathrm{Min}}$ is minimum air temperature (°C), ${T}_{\mathrm{Mean}}$ is mean air temperature (°C), ${T}_{\mathrm{range}}$ is temperature range (°C), ${T}_{\mathrm{Dew}}$ is dew point temperature (°C), ${H}_{\mathrm{Avg}}$ is average relative humidity (%), ${H}_{\mathrm{Range}}$ is range of relative humidity (%), $\mathrm{WS}$ is wind speed, $\mathrm{Ele}$ is elevation (m), $\mathrm{Pop}$ is total population.

The total dataset was divided into a 70:30 ratio, where 70% of observations were used as a training dataset and the rest were used for testing. The accuracy of estimation was evaluated in terms of *R*^2^, root mean square error (RMSE) and mean bias (MB).

All the analyses were done using R programs.

## Results and Discussion

**Fig. 3:**
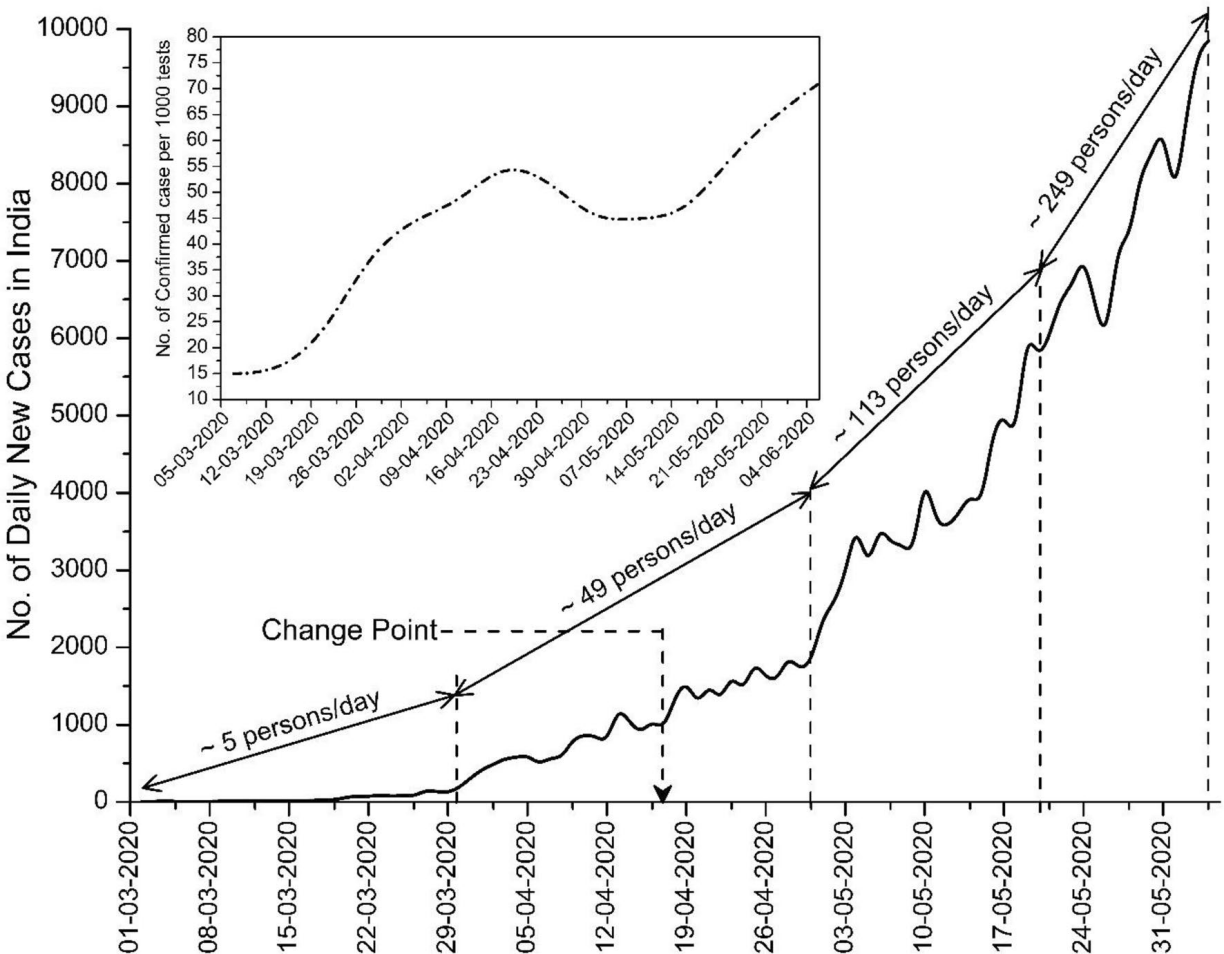
Trend of daily confirmed cases in India. The weekly trend of number of confirmed cases per 1000 tests are shown inset

**Fig. 4:**
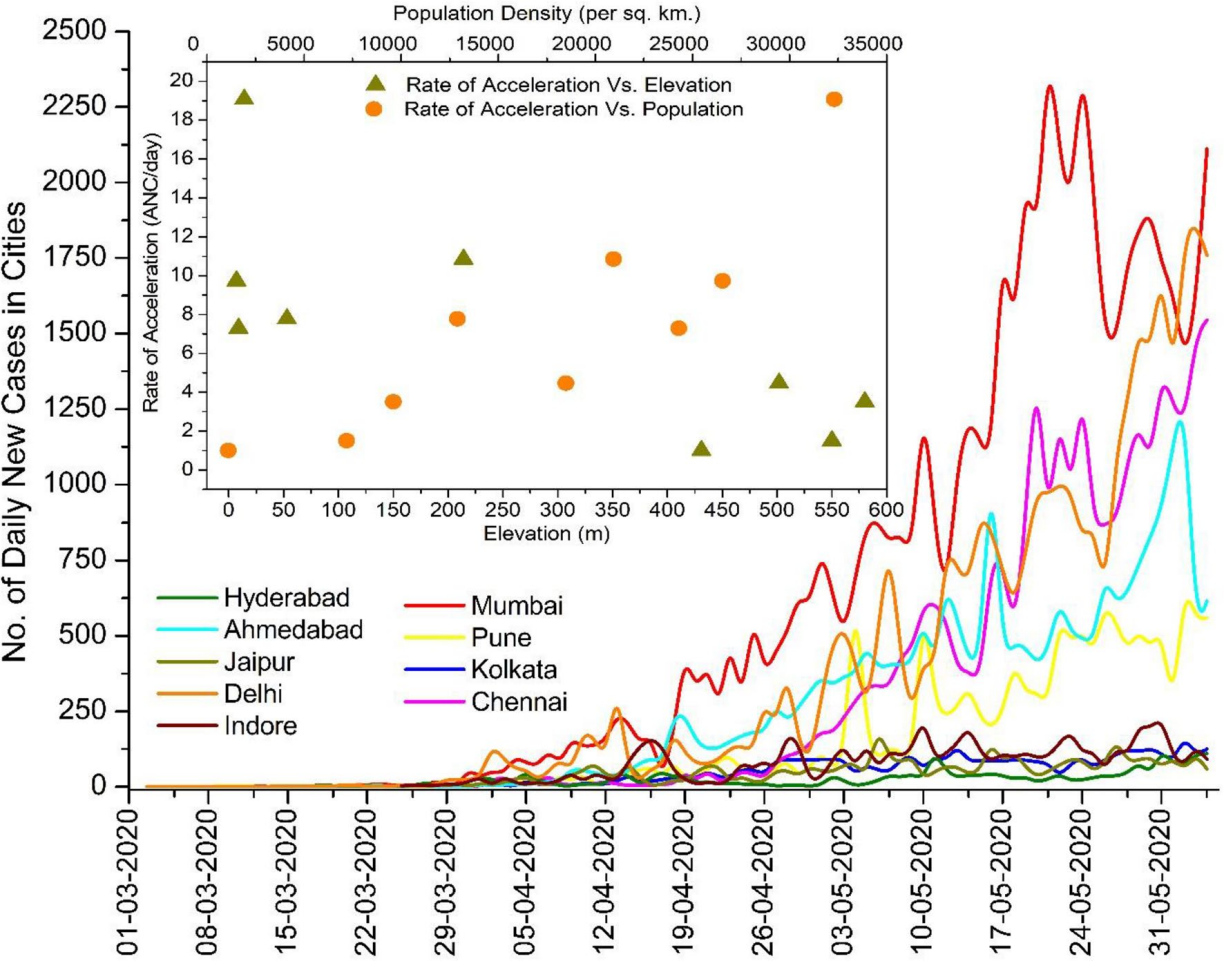
The daily trend of confirmed case in selected cities are shown. Inset is a scatter graph depicting the growth rate of transmission with respect to the population and elevation of those cities

Table 1 presents the results of the MK test, Sen’s slope and change point through the Pettitt test. All the results are found significant at *α* = 0.05 level, thus, there is significant change in the transmission pattern. The calculated Sen’s slope shows the rate of change in the count of additional new cases (ANC) in each day. For the whole country, an acceleration in confirmed cases of ~ 76 ANC/day was recorded from March 01 to June 04, 2020, whereas among the selected cities, Mumbai registered the highest rate of acceleration (~ 19 ANC/day). Analyses also revealed that the increasing rate of daily new cases accelerated abruptly all over the country after April 17, 2020, i.e., at the beginning of the 2nd phase of lockdown (April 15 to May 03, 2020). During the middle of April, a majority among the cities, namely, Ahmedabad, Chennai, Delhi, Indore, Kolkata, Pune and Mumbai registered the abrupt increase between April 12 and April 22, 2020. Only Hyderabad and Jaipur reported the abrupt increase during the 1st lockdown period (March 25 to April 14, 2020). Note too that all the major affected cities registered a 3 to 34-fold increase in transmission rate after the evaluated change point; explicitly, this incrementing rate was more than 20 times in Pune, Chennai and Ahmedabad after the estimated change point. The nationwide increase in transmission rate (case acceleration) was ~ 22 ANC/day before April 17 and afterward it amplified to ~ 174 ANC/day. Thus the entire country, and specifically the major affected cities, experienced an alarming rate of acceleration in the spread of COVID-19 during the lockdown period itself. Basically, the lockdown was implemented with a brief guideline of social distancing with an aim to reduce the occurrence of human-to-human transmission by avoiding gatherings at workplaces and at any other public places. Nationwide lockdowns resulted very effectively in reducing the growth rate of transmission in different countries across the world, such as China, Italy, France, Germany, United Kingdom (Gatto et al. 2020; Leung et al. 2020; Wurtzer et al. 2020). However, in India, the initial growth rate (acceleration) in infection up to March was approximately 5 ANC/day, and after that, the growth rate amplified multi-fold. Explicitly, the growth rate acceleration was ~ 49 ANC/day during April; it reached up to ~ 113 ANC/day during May 1 to May 20, and during May 21 to June 04, it was ~ 249 ANC/day (Fig. 3). One of major reasons behind the initial slow growth rate might be surmised to be that the original virus had been transmitted through an infected immigrant; moreover, very few tests conducted throughout the country during March (fewer than 10,000 tests/day). Analysis also reveals that within this 96 days study period, the percentage growth rate for the overall country was 10.79%, whereas among the selected cities, Mumbai had the highest growth rate (9.98%) while Jaipur had the smallest growth rate (5.67%). Basically, the growth rate was higher in the cities, which had a higher rate of acceleration in COVID-19 cases. On the other hand, the doubling time of COVID-19 cases for Mumbai (7.31 days) and Chennai (7.57 days) was very close to the countrywide situation (7.85 days). Hyderabad registered the slowest doubling time of 12.58 days. This shows that the daily count of COVID-19 cases was doubling in less than 8 days throughout the country, which is also a measure of the drastic adverse situation in India. From Fig. 3, it can be seen that an average of 53/1000 tests results were confirmed for infection during the entire study period. However, this positive rate was 35/1000 during the month of March; later, it rose to 44/1000 and 57/1000 during April 01–30, and May 01–June 04, 2020, respectively. This shows that the probability of detecting confirmed cases also increased each week, which may be evidence of community transmission. The trend of daily new cases in the major affected cities (Fig. 4) also indicates the large increase in daily transmission from May onwards. Figure 4 also shows that cities located at a lower elevation and having higher population registered a higher growth rate of transmission, thus agreeing with an early observation by (Gupta et al. 2020d). Of the five megacities in India, just three (Delhi, Mumbai, and Chennai) are the only cities where the count of daily infected cases exceeded 1200. One of the probable reasons behind such spikes in transmission rate might be the allowance to migrants to return to their native places, which instigated large crowds in various cities and gathering in transport hubs, as reported in many local and national newspapers, thus resulting in such an unforeseen increasing rate of transmission all over the country.

**Table 1:** Result of Mann–Kendall test, Sen's slope, Pettit test, growth rate and doubling time

Cities	M–K tau	Sen's slope	Change point	Slope before change point	Slope after change point	Growth rate (%)	Doubling time (days)
Ahmedabad	0.90^a^	7.77^a^	17-04-2020	0.46^a^	12.75^a^	8.85	8.31
Chennai	0.89^a^	8.73^a^	18-04-2020	0.91^a^	31.28^a^	8.89	7.57
Delhi	0.87^a^	10.85^a^	22-04-2020	1.78^a^	34.86^a^	8.67	8.53
Hyderabad	0.63^a^	1.47^a^	31-03-2020	0.51^a^	1.77^a^	5.87	12.68
Indore	0.70^a^	1.5^a^	14-04-2020	0.43^a^	1.49^a^	6.43	11.04
Jaipur	0.70^a^	1^a^	09-04-2020	0.19^a^	1.08^a^	5.67	12.25
Kolkata	0.82^a^	1.3^a^	17-04-2020	0.25^a^	1.43^a^	6.38	11.34
Mumbai	0.88^a^	19.08^a^	18-04-2020	2.79^a^	38.2^a^	9.98	7.31
Pune	0.83^a^	3.51^a^	12-04-2020	0.52^a^	10.63^a^	7.95	9.75
All over India	0.95^a^	76.11^a^	17-04-2020	21.55^a^	173.54^a^	10.79	7.85

^a^Statistics significant at 0.05 significance level

The Spearman correlation analysis (Table 2) shows that there were mostly significant but still predominantly low correlations between the number of daily new cases and the various weather conditions. Among the eight weather parameters, the correlation for *T*_range_ is non-significant over all time spans. Hence, the diurnal range of temperature is not significantly associated with the spread of COVID-19 cases in India. *H*_avg_ is associated significantly positively on the day of detection up to 10 days lag (i.e., when transmission presumably occurred). However, *H*_range_ is significantly negatively associated for 12–16 days prior to detection. Following the observations over the selected cities located in different geographical parts of the country, it is uncertain whether the higher humidity could reduce the infectivity of the coronavirus by reducing the suspension time of virus. This suggests that the role of humidity is quite complex and needs to be investigated further. On the other hand, all the temperature parameters (*T*_max_, *T*_min_, *T*_mean_, *T*_Dew_) are proportionately associated with COVID-19 transmission. The analysis also indicates that *T*_max_, *T*_min_, *T*_mean_, *T*_Dew_ and WS on the day of the detection have the lowest correlations, which improves up to its peak at a time lag of 14 days. In other words, the maximum, minimum, mean and dew point temperature along with wind speed at 14 days prior to detection are closely allied with the number of infections. This suggests the interesting inference that weather conditions 14 days prior to the detection of infections had provided favorable conditions for virus transmutability. Surprisingly, perhaps, *T*_min_ is found to be better related than *T*_mean_* T*_max_* T*_Dew_. Therefore, places with higher minimum temperature are more susceptible to COVID-19 transmission in India. A significant positive correlation between WS and daily transmission at a lag of 14 days infers that the virus might be able to transmigrate in high winds. Since most of the weather parameters are better correlated with the daily confirmed cases at a time lag of 14 days, this indicates an approximate incubation period of around 14 days for this disease in the Indian scenario. Therefore, considering the lag period of 14 days, the correlation analysis for each selected city (Table 3) shows that cities located away from the coast such as Delhi, Indore and Jaipur have better association with temperature parameters (*T*_max_, *T*_min_, *T*_mean_, *T*_Dew_) than the coastal cities such as Mumbai, Chennai and Kolkata. However, WS is relatively better correlated with COVID-19 cases in coastal cities than in the other cities. Interestingly, RH_Avg_ and RH_Range_ are also significantly related with COVID-19 cases in coastal cities only, while cities located in the interior did not exhibit any significant correlation. That is why on the country-wide scale, correlations between COVID-19 cases and RH parameters were reporting as non-significant. Hence, a higher humidity with a higher wind speed could be favourable for virus transmissibility; while a higher temperature might favor virus transmission in semi-arid and interior areas. This also suggests that the geographical location of the cities plays a crucial role in the association of weather parameters with COVID transmission, which makes this interrelationship even more complex.

**Table 2:** Result of Spearman's correlation test in different time frames for all over India

Parameters	On that day	7 days ago	10 days ago	12 days ago	14 days ago	16 days ago
Maximum temperature	0.161^a^	0.198^a^	0.231^a^	0.336^a^	0.347^a^	0.272^a^
Minimum temperature	0.244^a^	0.285^a^	0.319^a^	0.417^a^	0.436^a^	0.351^a^
Mean temperature	0.199^a^	0.248^a^	0.287^a^	0.409^a^	0.430^a^	0.337^a^
Temperature range	− 0.032	− 0.043	− 0.066	− 0.064	− 0.075	− 0.054
Dew point temperature	0.222^a^	0.235^a^	0.261^a^	0.238^a^	0.269^a^	0.238^a^
Relative humidity	0.128^a^	0.10^a^	0.10^a^	0.002	0.008	0.034
Humidity range	− 0.016	− 0.024	− 0.056	− 0.13^a^	− 0.149^a^	− 0.095
Wind speed	0.108^a^	0.133^a^	0.163^a^	0.221^a^	0.255^a^	0.193^a^

^a^Statistics significant at 0.05 significance level

**Table 3:** Result of Spearman's correlation test in each cities considering a lag of 14 days only

Parameters	Ahmedabad	Chennai	Delhi	Hyderabad	Indore	Jaipur	Kolkata	Mumbai	Pune
Maximum temperature	0.46^a^	0.23^a^	0.51^a^	0.31^a^	0.53^a^	0.51^a^	0.15^a^	0.22^a^	0.27^a^
Minimum temperature	0.51^a^	0.38^a^	0.56^a^	0.34^a^	0.58^a^	0.55^a^	0.17^a^	0.29^a^	0.29^a^
Mean temperature	0.45^a^	0.29^a^	0.50^a^	0.33^a^	0.51^a^	0.54^a^	0.14^a^	0.28^a^	0.23^a^
Temperature range	− 0.06	− 0.19^a^	− 0.07	− 0.08	− 0.07	− 0.13	− 0.26^a^	− 0.23^a^	− 0.12
Dew point temperature	0.27^a^	0.25^a^	0.30^a^	0.28^a^	0.29^a^	0.17^a^	0.24^a^	0.26^a^	0.25^a^
Relative humidity	− 0.18	0.21^a^	− 0.39^a^	− 0.50^a^	− 0.13	− 0.20	0.45^a^	0.46^a^	− 0.17
Humidity range	− 0.07	− 0.18^a^	− 0.11	− 0.08	− 0.11	− 0.12	− 0.24^a^	− 0.22^a^	− 0.09
Wind speed	0.32^a^	0.49^a^	0.21^a^	0.20^a^	0.13^a^	0.17^a^	0.32^a^	0.37^a^	0.24^a^

^a^Statistics significant at 0.05 significance level

**Fig. 5:**
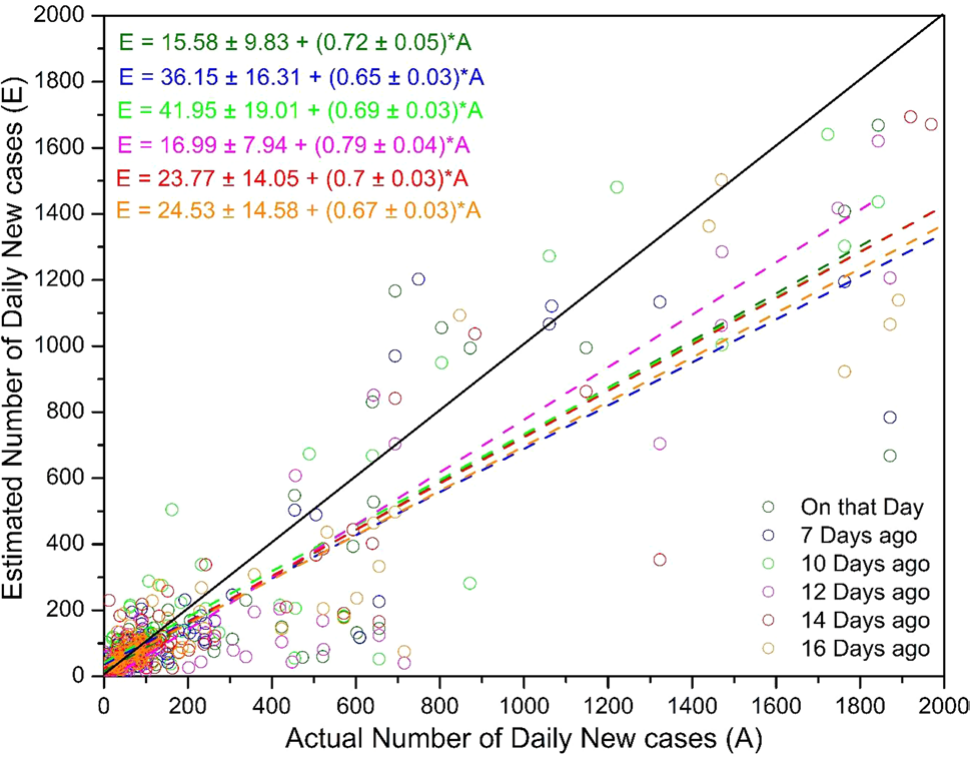
Validation of SVM-based regression model for estimating daily transmission

Figure 5 and Table 4 show the validation of estimated daily confirmed cases for all time spans using the non-linear multivariate Support Vector Regression Model with RBF kernel.

**Table 4:** Result of validation of SVM-based regression for estimating daily transmission

	*R*^2^	RMSE	MB
On that day	0.6414	199.2929	− 48.1804
7 days ago	0.7015	202.1743	− 40.0377
10 days ago	0.8286	223.1949	− 42.0560
12 days ago	0.8503	186.0126	− 66.9880
14 days ago	0.8680	178.3891	− 43.6459
16 days ago	0.8714	202.2428	− 60.0658

**Fig. 6:**
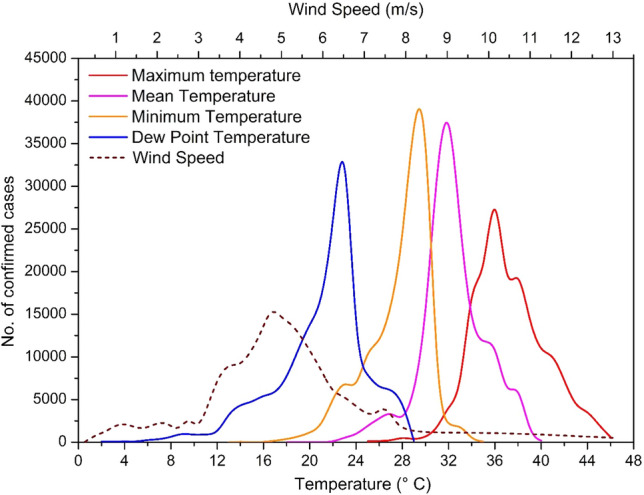
Influence of weather parameters on count of confirmed cases with a lag of 12–16 days

The model performance in terms of *R*^2^, RMSE, and MB are presented in Table 4. This shows that the SVM-based regression model is very efficient in establishing the complex relationship between the different weather parameters and the daily transmission of COVID-19. However, it underestimates the prevalence of very high values (> 1200 cases). Hence, we may conclude that no single weather parameter is enough to linearly correlate with the daily transmission. Instead, a non-linear multivariate approach is needed to estimate the daily transmission in India with high accuracy. Correlation analysis has evidently revealed a relatively higher degree of association with daily new cases for most of the parameters only when the time lag of 14 days is taken into consideration. The SVM-based regression model also performs remarkably well with a time lag of 12–16 days. Together, these suggest a typical incubation period of 12–16 (14 ± 02) days for COVID-19 transmission in India. To better understand the influence of varying weather conditions, the response curves of each significant parameter to the cumulative confirmed cases were calculated and are shown in Fig. 6, which reveals that cumulative COVID cases are very high only during a certain range of temperature and wind speed. Approximately 75% of the total confirmed cases were registered when the *T*_Max_, *T*_Mean_, *T*_Min_, *T*_Dew_, and WS parameters 12–16 days previously vary within a range of 33.6–41.3 °C, 29.8–36.5 °C, 24.8–30.4 °C, 18.7–23.6 °C, and 4.2–5.75 m/s, respectively. Briefly, over the selected cities, there is quite large variation in daily weather conditions, hence, there is a broad range in the values of each weather parameter, among which a narrow range is highly favorable for virus transmission, as revealed by this study. This also suggests why the linear univariate correlation was so low for each of the parameters. Together, these give an idea of the favorable weather conditions for such transmission in India. In other words, the areas in India that experienced such weather patterns are those most likely to have been affected by this disease.

## Conclusions

Unlike most studies, the present study investigated the impact of various weather parameters which include maximum, minimum, mean, and dew point temperature, temperature range, average humidity, humidity range and wind speed on the same day, as well as with time-lags of 7, 10, 12, 14, and 16 days prior to detection of the confirmed cases of COVID-19 in the Indian context. Additionally, the daily trends of confirmed cases in nine of the most affected cities in India, along with a comparison of the entire country, have also been inspected in this study. The analyses revealed that the count of confirmed cases is not well correlated with any individual meteorological parameter because simple correlation depicts a linear relationship only. Rather than that, COVID-19 cases are significantly associated with a very certain range of temperature parameters and wind speed. Thus, much better than linear correlation, the non-linear SVM-based regression approach efficiently resolved this complex association and was able to estimate the daily cases of infection quite accurately with the help of the daily weather inputs. However, the positive correlation between daily transmission and air temperature, as well as wind speed, indicates that the daily transmission in highly populated areas in India has consequently increased during the current summer days of 2020. An approximate incubation period of 14 ± 02 days can also be identified from the data, which is a little longer than what WHO had estimated early in March. Therefore, in the prevailing weather conditions in India, the SARS-CoV-2 can be disseminated into the surrounding environment for around 2 weeks after being ingested from any other infected source.

The COVID-19 pandemic has resulted in a state of recrudescence in India. The daily confirmed cases have been rising at an acceleration rate of ~ 76 ANC/day since March 2, 2020 with a doubling rate of 7.85 days. This rate of acceleration all over the country reached approximately 249 ANC/day during the starting of June. Initially, 14 out of each 1000 tests revealed positive results during the first week of March, but the positive test rate escalated to 71/1000 tests in the first week of June. On the other hand, reduced strictness in subsequent phases of lockdowns, along with the allowing of interstate migration, had inevitably caused an easy pathway for transmission, hence resulting in an intractable circumstance all over the country. The cities with larger populations are cataloguing a higher rate of increase in daily cases. Moreover, a step-change in the rising trend over all the major affected cities has also been noted during mid-April, i.e., at the boundary between the first and second lockdowns. This signifies that the imposed lockdown was unsuccessful in reducing the COVID-19 transmission in India, unlike in e.g., South Korea, Japan, and Iran. Nonetheless, this study has limitations, since we were unable to include many other major affected cities due to lack of meteorological data availability. Moreover, the number of immigrants from abroad or other cities who were quarantined was not available; these might have enhanced the exactitude of the current analysis.
